# The transgenic IG-DMR sequence of the mouse *Dlk1-Dio3* domain acquired imprinted DNA methylation during the post-fertilization period

**DOI:** 10.1186/s13072-023-00482-x

**Published:** 2023-02-17

**Authors:** Hitomi Matsuzaki, Shokichi Sugihara, Keiji Tanimoto

**Affiliations:** 1 Faculty of Life and Environmental Sciences, Life Science Center for Survival Dynamics, Tsukuba Advanced Research Alliance (TARA), University of Tsukuba, Tennoudai 1-1-1, Tsukuba, Ibaraki 305-8577 Japan; 2 Graduate School of Life and Environmental Sciences, University of Tsukuba, Tsukuba, Ibaraki 305-8577 Japan

**Keywords:** Genomic imprinting, Imprinting control region, DNA methylation

## Abstract

**Background:**

Allele-specific methylation of the imprinting control region (ICR) is the molecular basis for the genomic imprinting phenomenon that is unique to placental mammals. We previously showed that the ICR at the mouse *H19* gene locus (*H19* ICR) was unexpectedly established after fertilization and not during spermatogenesis in transgenic mice (TgM), and that the same activity was essential for the maintenance of paternal methylation of the *H19* ICR at the endogenous locus in pre-implantation embryos. To examine the universality of post-fertilization imprinted methylation across animal species or imprinted loci, we generated TgM with two additional sequences.

**Results:**

The rat *H19* ICR, which is very similar in structure to the mouse *H19* ICR, unexpectedly did not acquire imprinted methylation even after fertilization, suggesting a lack of essential sequences in the transgene fragment. In contrast, the mouse IG-DMR, the methylation of which is acquired during spermatogenesis at the endogenous locus, did not acquire methylation in the sperm of TgM, yet became highly methylated in blastocysts after fertilization, but only when the transgene was paternally inherited. Since these two sequences were evaluated at the same genomic site by employing the transgene co-placement strategy, it is likely that the phenotype reflects the intrinsic activity of these fragments rather than position-effect variegation.

**Conclusions:**

Our results suggested that post-fertilization imprinted methylation is a versatile mechanism for protecting paternal imprinted methylation from reprogramming during the pre-implantation period.

**Supplementary Information:**

The online version contains supplementary material available at 10.1186/s13072-023-00482-x.

## Background

**Fig. 1:**
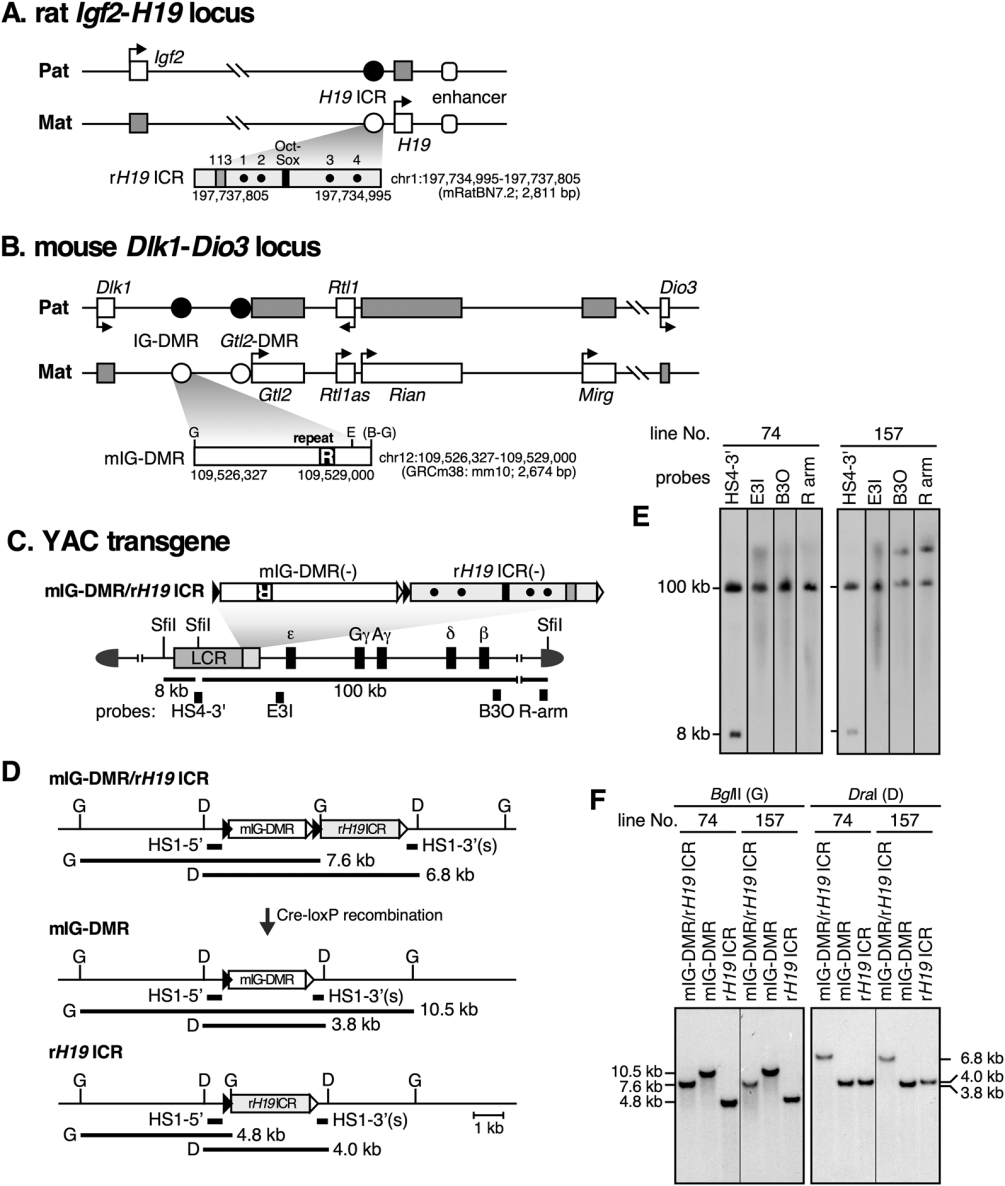
Generation of rat *H19* ICR and mouse IG-DMR transgenic loci at the identical chromosomal site. **A** Structure of the rat *Igf2-H19* gene locus. Monoallelic expression of paternal *Igf2* and maternal *H19* genes depends on the shared 3' enhancer and methylation state of the *H19* ICR, that is methylated (solid circle) and unmethylated (open circle) at the paternal and maternal alleles, respectively. In the enlarged rat *H19* ICR map (r*H19* ICR), CTCF and Sox/Oct binding sites are indicated by dots (1–4) and a solid box, respectively. The 113-bp sequence homologous to the “mouse 118-bp sequence” is denoted as gray box. **B** Structure of the mouse *Dlk1-Dio3* domain, in which three protein-coding genes, i.e., *Dlk1*, *Rtl1*, and *Dio3* are monoallelically expressed from the paternal allele, while multiple noncoding transcripts, such as *Gtl2*, *Rtl1as*, *Rian*, and *Mirg*, are maternally expressed. Active genes on each allele are represented by white rectangles with their transcriptional directions shown by arrows. IG-DMR is methylated (solid circle) and unmethylated (open circle) at the paternal and maternal alleles, respectively. Gtl2-DMR is a secondary DMR. The structure of mouse IG-DMR (mIG-DMR) sequences used in this study is enlarged beneath the map. **C** Structure of the 150-kb human β-globin locus YAC, in which LCR and β-like globin genes are indicated by shaded and solid boxes, respectively. Each of the mIG-DMR and r*H19* ICR fragment was floxed by a pair of loxP variants [loxP5171 (solid triangle) and loxP2272 (open)], tandemly arranged and introduced 3' to the LCR for employing co-placement strategy. The expected *Sfi*I restriction enzyme fragments (thick lines) generated from the YAC transgene and probe locations (filled rectangles) are shown beneath the map. **D** In vivo Cre-loxP recombination to derive mIG-DMR or r*H19* ICR TgM. Recombination between two loxP5171 sites (solid) in the parental mIG-DMR/r*H19* ICR transgene, for example, would generate r*H19* ICR allele, during which one of the loxP2272 sites (open) is concomitantly removed to prevent further recombination. **E** Long-range structural analysis of the YAC transgene prior to Cre-loxP excision reaction. DNA from thymic cells was digested with *Sfi*I in agarose plugs and separated by pulsed-field gel electrophoresis, and Southern blots were hybridized separately to probes. **F** Tail DNA from parental and daughter YAC-TgM sublines was digested with *Bgl*II (G: left) or *Dra*I (D: right) and analyzed by Southern blotting using the probes shown in D (HS1-5' and HS1-3' probes for *Bgl*II and *Dra*I digestion, respectively) to confirm correct recombination events

Epigenetics refers to a process wherein regulation of genome function is governed by various chromatin modifications that are inherited over cell divisions or generations. Examples include genomic imprinting, X chromosome inactivation, and regulation of cell lineage-specific gene expression. Genomic imprinting is a mono-allelic gene expression mechanism in placental mammals in which certain genes are expressed from only one of the parental alleles [1–5]. Many of the imprinted genes form clusters on the genome and play pivotal roles in embryonic growth, placental function, and other processes. For example, at the *Igf2* (insulin-like growth factor 2)/*H19* (noncoding RNA) gene domain, the paternally expressed *Igf2* gene promotes embryonic growth while the maternally expressed *H19* gene inhibits cell proliferation. These genes are ~ 100 kilobase (kb) pairs apart and their unique expression pattern is regulated by the imprinting control region (*H19* ICR) present 2–4 kb upstream of the transcription start sites of the *H19* gene (Fig. 1A; [6–9]).

Genomic regions whose DNA methylation states differ between parental alleles are called differentially methylated regions (DMRs). In genomic imprinting, allele-specific deposition of DNA methylation is thought to serve as an epigenetic mark to distinguish the parental origin of the alleles and to control imprinted gene expression [3, 5]. *H19* ICR, a typical DMR, is methylated in sperm but unmethylated in oocytes, and this differential methylation state is maintained beyond fertilization. Another example of a DMR is the IG (intergenic)-DMR present in the *Dlk1*/*Dio3* gene domain, in which imprinted expression of *Dlk1*/*Rtl1*/*Dio3*/*Gtl2*/*Rian*/*Mirg* transcripts occurs due to differential methylation of the IG-DMR (Fig. 1B; [10]).

Among DMRs, sequences whose methylation states are established during gametogenesis are called gametic (gDMRs) or primary DMRs, whereas regions that become differentially methylated after fertilization, based on the methylation state of the *cis*-linked primary DMR or the transcriptional state of the nearby imprinted gene, are called secondary DMRs. Once established in germ cells, most gDMRs are subjected to passive DNA demethylation associated with multiple cell divisions and DNA replication after fertilization, as well as active demethylation, governed by TET enzymes, as embryonic reprogramming proceeds. Even in these circumstances, some DMRs (including ICRs) must maintain their DNA methylation states for executing correct genomic function throughout life. Only three ICRs, including *H19* ICR and IG-DMR, acquire DNA methylation in their intergenic region during spermatogenesis, whereas over 20 ICRs acquire methylation in their promoter region during oogenesis, and both types of ICRs are maintained after fertilization to regulate genomic imprinting. Thus, in addition to the process whereby allele-specific methylation is established in the germline, the mechanisms by which it is maintained during early embryogenesis have been increasingly recognized to play important roles in its inheritance [11, 12].

To understand the molecular mechanisms underlying the allele-specific establishment and maintenance of DNA methylation, we previously generated TgM carrying the mouse *H19* ICR and found that the sequence was not methylated in sperm, and instead acquired DNA methylation immediately after fertilization, but only when paternally inherited. We termed this unexpected phenomenon “post-fertilization imprinted DNA methylation” [13, 14]. Because of the parental origin of the *H19* ICR and the fact that DNA methylation was specifically deposited on the paternal allele, we considered that an epigenetic signature other than DNA methylation is set within the *H19* ICR in sperm and is then recognized by DNA methylation machinery after fertilization. The intrinsic ability of the mouse *H19* ICR to establish imprinted methylation has also been demonstrated at other gene loci (*alpha fetoprotein* and *IgH*) [15, 16]. Then, we identified a *cis* sequence (118 bp) that is essential for the activity in TgM, deleted the sequence from the endogenous *Igf2*/*H19* locus, and found a marked decrease in the methylation level of the endogenous paternal *H19* ICR sequence after fertilization, with no effect on its methylation state in sperm [17, 18]. Therefore, the post-fertilization imprinted DNA methylation activity seemed essential for the pre-implantation maintenance of paternal methylation of the endogenous *H19* ICR. Apart from our findings, the Zfp57 and Zfp445 proteins have been reported to be involved in the maintenance of the hypermethylation state of both paternally and maternally methylated DMRs, including the *H19* ICR and IG-DMR, at least during the post-implantation period [19–21]. If these proteins play a role in pre-implantation embryos, how the Zfp57/445-dependent mechanism and the one governed by the 118-bp sequence we discovered share their roles has yet to be investigated.

In post-implantation embryos, cell fate decisions are accompanied by de novo methylation of the entire genome, but even under these circumstances, the hypomethylation state of some DMRs, including the maternal *H19* ICR and IG-DMR, must be maintained. Whereas it has been established that the CTCF and Sox/Oct transcription factors are essential for the persistent hypomethylation state of the *H19* ICR [22–25], they cannot be responsible for this state in the IG-DMR because hypomethylation is maintained in post-implantation embryos despite the apparent absence of CTCF factor binding within the region [26]. Thus, the underlying mechanism has not yet been fully elucidated.

As mentioned earlier, we identified the 118-bp sequence that is essential for post-fertilization imprinted methylation of the mouse *H19* ICR during the pre-implantation period [17, 18, 27]. We further demonstrated that this sequence, together with the CTCF and Sox/Oct binding sequences, could confer imprinted methylation activity to the non-imprinted lambda DNA sequence both in transgenic [27] and endogenous genomic contexts [28].

Although the 118-bp sequence we found in the mouse *H19* ICR plays a central role in genomic imprinting at the *Igf2*/*H19* locus, the *cis* motif(s) and related binding factors that are responsible for this activity have yet to be determined. To obtain clues about the *cis* motifs, we generated TgM with a human *H19* ICR (8.8 kb) fragment and found that a paternally inherited transgenic sequence preferentially acquired DNA methylation after fertilization [29]. Although it was expected that the human *H19* ICR would contain a sequence orthologous to the mouse 118-bp sequence, we could not identify *cis* DNA motifs that were important for executing post-fertilization paternal DNA methylation because their sequence identity was not high enough. In this study, therefore, we employed the rat *H19* ICR sequence, which is significantly homologous to the mouse sequence, and attempted to identify sequences important for imprinted methylation. The overall structure of the rat *Igf2*/*H19* locus and its *H19* ICR sequence are both similar to those in mice (Fig. 1A and Additional file 1: Fig. S1A; [8, 30]. The 2.8-kb test fragment contained a 113-bp sequence that apparently corresponded to the mouse 118-bp sequence, as well as four CTCF and one Sox/Oct binding sequences that protect the maternal *H19* ICR sequence from genome-wide DNA methylation after the implantation period (Additional file 1: Fig. S1B, C).

In addition, to further clarify the universality of post-fertilization imprinted methylation activity in the genomic imprinting mechanism, we employed the 2.7-kb mouse IG-DMR sequence. As mentioned earlier, the mIG-DMR is one of three ICRs that is methylated in sperm, and its methylation state is maintained after fertilization. It carries a repeat sequence (Fig. 1B) that is well conserved across species [31], and deletion of this sequence results in loss of DNA methylation in the paternal mIG-DMR after fertilization [26, 32]. Thus, the sequence may also be involved in the post-fertilization maintenance of paternally inherited DNA methylation.

In this study, to examine if these two paternally methylated ICR fragments undergo post-fertilization methylation imprinting, we generated corresponding yeast artificial chromosome (YAC) TgM by using a transgene co-placement strategy, a rigorous methodology employed in TgM experiments [33], and carefully examined their DNA methylation states.

## Results

### Generation of YAC TgM harboring mIG-DMR/r*H19* ICR sequences

In genomic imprinting studies, the use of mammalian models is essential to assess how chromatin modifications that occur in parental germ cells affect the somatic cells of the next generation. We thus generated TgM to examine the activity of two test fragments, the rat *H19* ICR (r*H19* ICR; Fig. 1A) and mouse IG-DMR (mIG-DMR; Fig. 1B). One drawback of this experimental system is position-effect variegation, in which behavior of (even identical) transgenic fragments differs significantly depending on the positions of their integration sites in the mouse genome. To circumvent this problem, we embedded each test fragment into a 150-kb human β-globin YAC to reduce the influence of the chromatin state around the transgene insertion site (Fig. 1C; [13]). Furthermore, we used a transgene co-placement strategy to reduce the labor necessary to generate multiple strains of TgM, as well as to allow comparison, if necessary, between the activity of different fragments at the identical genomic position (Fig. 1D).

Each test fragment was flanked by two different loxP sequences (loxP5171 and 2272) that were aligned in tandem and inserted downstream of the locus control region (LCR) of the human β-globin YAC (Fig. 1C). Purified YAC DNA was microinjected into fertilized mouse eggs to generate TgM. Tail-tip DNA of newborn pups was used to screen TgM and to perform copy number analysis by PCR (data not shown). Then, high-molecular-weight DNA was prepared from the thymus of candidate TgM that potentially carried a single copy of the YAC transgene. Long-range Southern blot analysis (Fig. 1E) revealed that two strains of transgenic mouse (lines 74 and 157) carried at least one intact transgene copy. Although the 3' portion of the YAC sequence (downstream of the β-globin gene) was additionally retained in line 157, we assumed this did not affect the methylation state of the test fragment inserted 3ʹ to the LCR. Next, we crossed parental YAC TgM (mIG-DMR/r*H19* ICR) with Zp3-Cre TgM expressing the Cre enzyme in the egg to induce an in vivo Cre-loxP recombination reaction (Fig. 1D). Southern blot analysis of tail-tip DNA from the next and subsequent generations of mice showed that sublines of TgM that carried only the mIG-DMR or r*H19* ICR sequences were established for both lines 74 and 157 (Fig. 1F).

### DNA methylation analysis of the r*H19* ICR transgene by methylation-sensitive restriction enzyme-coupled qPCR (qMethyl-PCR)

**Fig. 2:**
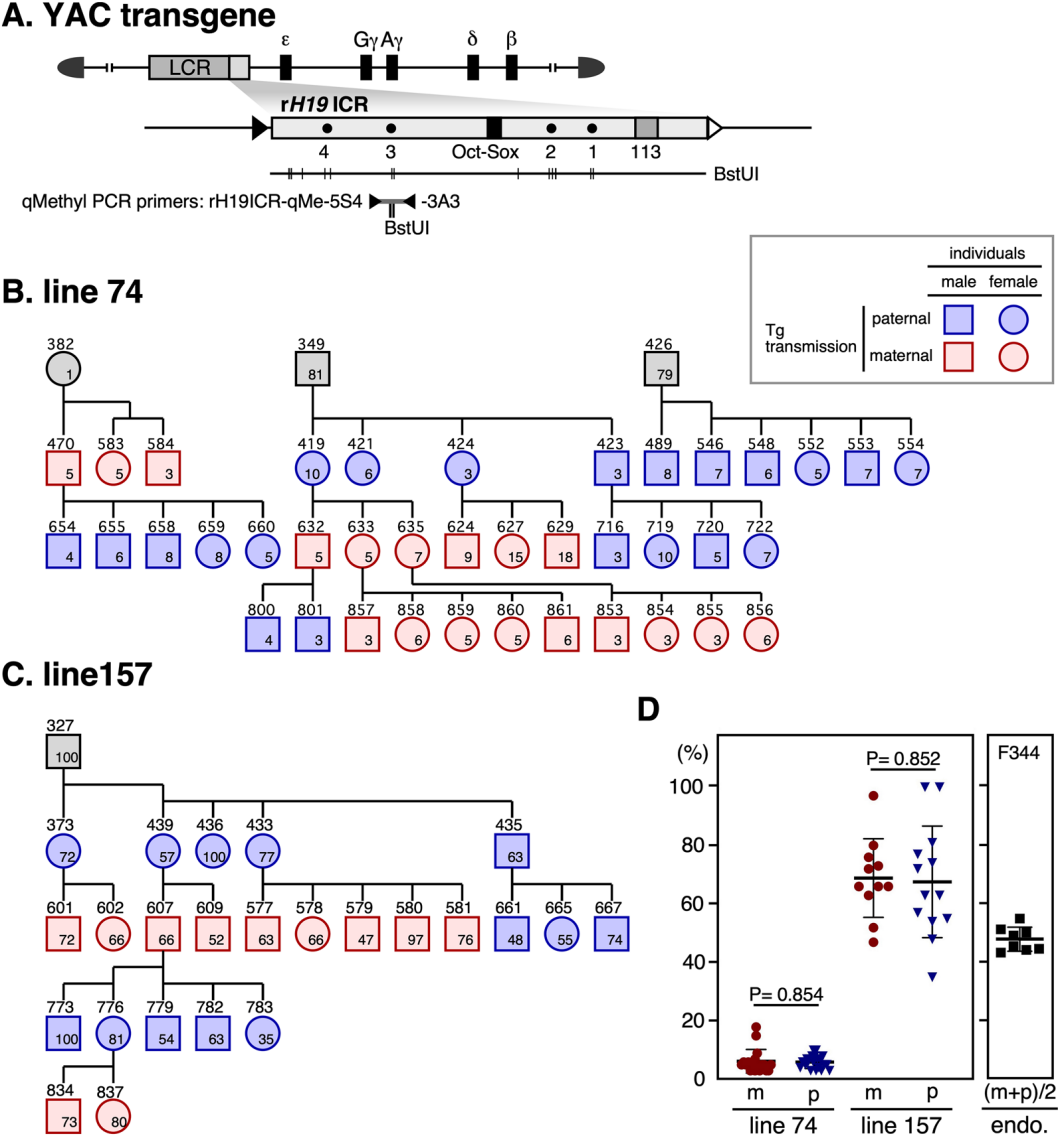
Methylation analysis of r*H19* ICR transgene by qMethyl-PCR. **A** Structure of the r*H19* ICR transgene. The region analyzed by qMethyl-PCR (B–D) is shown beneath the enlarged r*H19* ICR map. The locations of DNA methylation-sensitive *Bst*UI sites in the r*H19* ICR, as well as those in the qMethyl-PCR target sequences, are shown by verical lines. **B**, **C** Pedigree (non-transgenic individuals are not shown) and methylation level of the *Bst*UI sites at around the CTCF site 3. Squares and circles (with their identification numbers shown above each object) represent male and female individuals, respectively. Blue and red colors indicate those inheriting the transgene paternally and maternally, respectively. Numbers in the objects represent DNA methylation levels of the transgenes determined by qMethyl-PCR (average of two technical replicates). **D** Methylation levels of the paternally (p, blue) or maternally (m, red) inherited transgenes were compared and mean ± SD values for each line are graphically depicted. P values < 0.05 are considered significantly different. As a control, the methylation level of the same sequence at the endogenous locus was analyzed without discriminating parental alleles (the expected value is around 50%, as shown in Fig. 3B)

We next bred the r*H19* ICR TgM and examined the methylation state of the tail-tip DNA of the pups (1–2 weeks old) using qMethyl-PCR. Since imprinted methylation of the mouse *H19* ICR transgene was observed in almost the entire 2.9-kb transgene fragment in tail somatic cell DNA, and because the maternally inherited *H19* ICR is known to be protected from genome-wide de novo methylation by a CTCF-dependent mechanism after implantation, we analyzed a region around the CTCF binding site 3 in rat *H19* ICR (Fig. 2A). In line 74, the methylation level in this region of the transgene was uniformly low irrespective of whether the transgene was parentally or maternally inherited (Fig. 2B, D). While the methylation level in the same region was relatively high in line 157, it was also uniform regardless of parent of origin (Fig. 2C, D). The difference in methylation levels between the two lines is probably attributable to the position-effect variegation of the transgene.

### DNA methylation analysis of the r*H19* ICR transgene by bisulfite sequencing

**Fig. 3:**
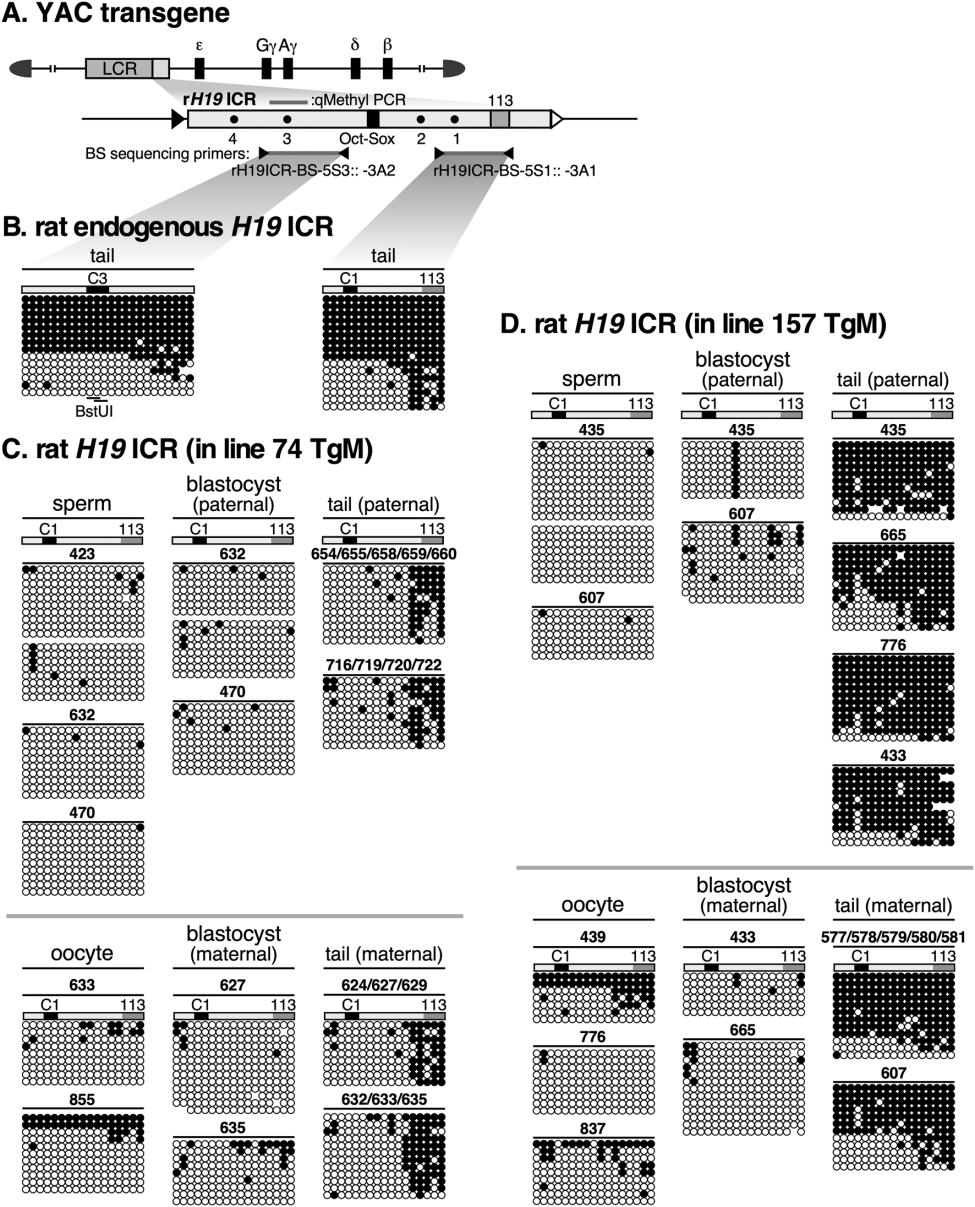
Bisulfite sequencing analysis of r*H19* ICR transgene. **A** Enlarged map of the r*H19* ICR transgene. The region (gray horizontal bar) analyzed by qMethyl-PCR in Fig. 2 is shown above the enlarged map. The regions amplified by PCR for BS sequencing and the primer sets used are shown beneath the enlarged map. **B** Genomic DNA from tail-tip somatic cells of rat was subjected to BS sequencing. Each horizontal row represents a single DNA template molecule. Methylated and unmethylated CpG motifs are shown as filled and open circles, respectively. Positions of CTCF binding sites 1, 3 (C1 and C3) and 113-bp region are shown by solid and gray rectangles, respectively. The CpG motifs in the *Bst*UI recognition site are marked by horizontal lines. **C**, **D** Genomic DNAs from germ cells (sperm or oocyte), blastocyst, and tail-tip somatic cells of TgM (lines 74 and 157 in C and D, respectively) inheriting the transgene either paternally (upper panels) or maternally (lower) were subjected to BS sequencing. ID numbers shown above each block correspond to those shown in Fig. 2B, C

Next, we examined the DNA methylation state of the CpG sites in the r*H19* ICR using bisulfite sequencing (Fig. 3A). We first used rat genomic DNA to assess the endogenous rat *H19* ICR at its 5ʹ region (around the 113-bp sequence and CTCF site 1) and 3ʹ region (around the region analyzed by qMethyl-PCR in Fig. 2), and found bimodal distributions of hyper- and hypomethylated strands, except for the region around the 113-bp sequence, which was consistently methylated (Fig. 3B).

Then, we analyzed the transgene sequence around the 113-bp sequence and CTCF site 1. In both lines 74 and 157 (Fig. 3C, D), the transgene was almost devoid of methylation in sperm and eggs, and in addition neither of the alleles was methylated in blastocysts, indicating that post-fertilization methylation imprinting does not take place in the r*H19* ICR transgene. The CpG sites around the 113-bp sequence became methylated in both lines 74 and 157 after implantation (i.e., in the tail), regardless of whether they were paternally or maternally inherited (Fig. 3C, D). This suggested that these sites were subjected to genome-wide de novo DNA methylation. On the other hand, the region around CTCF site 1 in line 74 remained hypomethylated regardless of parental origin (Fig. 3C), which is probably due to the antagonistic activity of CTCF against de novo DNA methylation.

### DNA methylation analysis of the mIG-DMR transgene by qMethyl-PCR

**Fig. 4:**
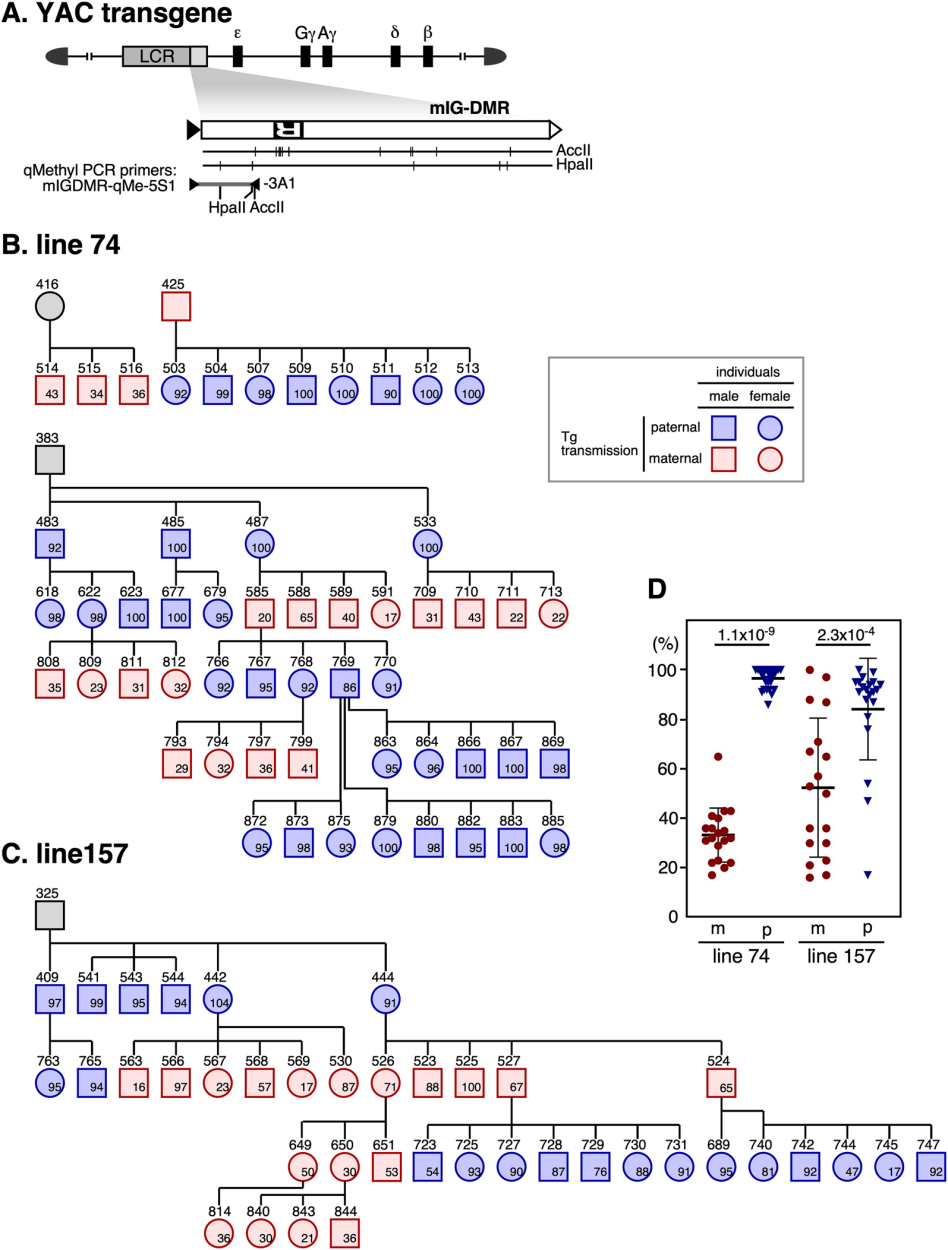
Methylation analysis of mIG-DMR transgene by qMethyl-PCR. **A** Structure of the mIG-DMR transgene. The region analyzed by qMethyl-PCR (**B**–**D**) is shown beneath the enlarged mIG-DMR map. The locations of DNA methylation-sensitive *Hpa*II and *Acc*II sites in the mIG-DMR, as well as the qMethyl-PCR target sequences, are shown by vertical lines. **B**, **C** Pedigree (non-transgenic individuals are not shown) and methylation level of the *Hpa*II/*Acc*II sites adjacent to the repeat sequence. The meaning of each object is same as those described in the legends to Fig. 2B, C. **D** Methylation levels of the paternally (p, blue) or maternally (m, red) inherited transgenes were compared and mean ± SD values for each line are graphically depicted. P values < 0.05 are considered significantly different

We next bred IG-DMR TgM and used qMethyl-PCR to analyze the methylation level of mouse tail-tip DNA at 1 − 2 weeks of age. To analyze the vicinity of the repeat sequence that is important for imprinted methylation activity, we designed a PCR primer set that specifically amplifies the 3ʹ (at the endogenous locus) side of the transgenic IG-DMR sequence (Fig. 4A). The results showed that in both lines 74 and 157, the DNA methylation level was significantly higher when the transgene was inherited from the father (Fig. 4B–D). Importantly, the DNA methylation state of the transgene was reset each time it was passed to the next generation, which is one of the key features of genomic imprinting [34].

### DNA methylation analysis of the mIG-DMR transgene by bisulfite sequencing

**Fig. 5:**
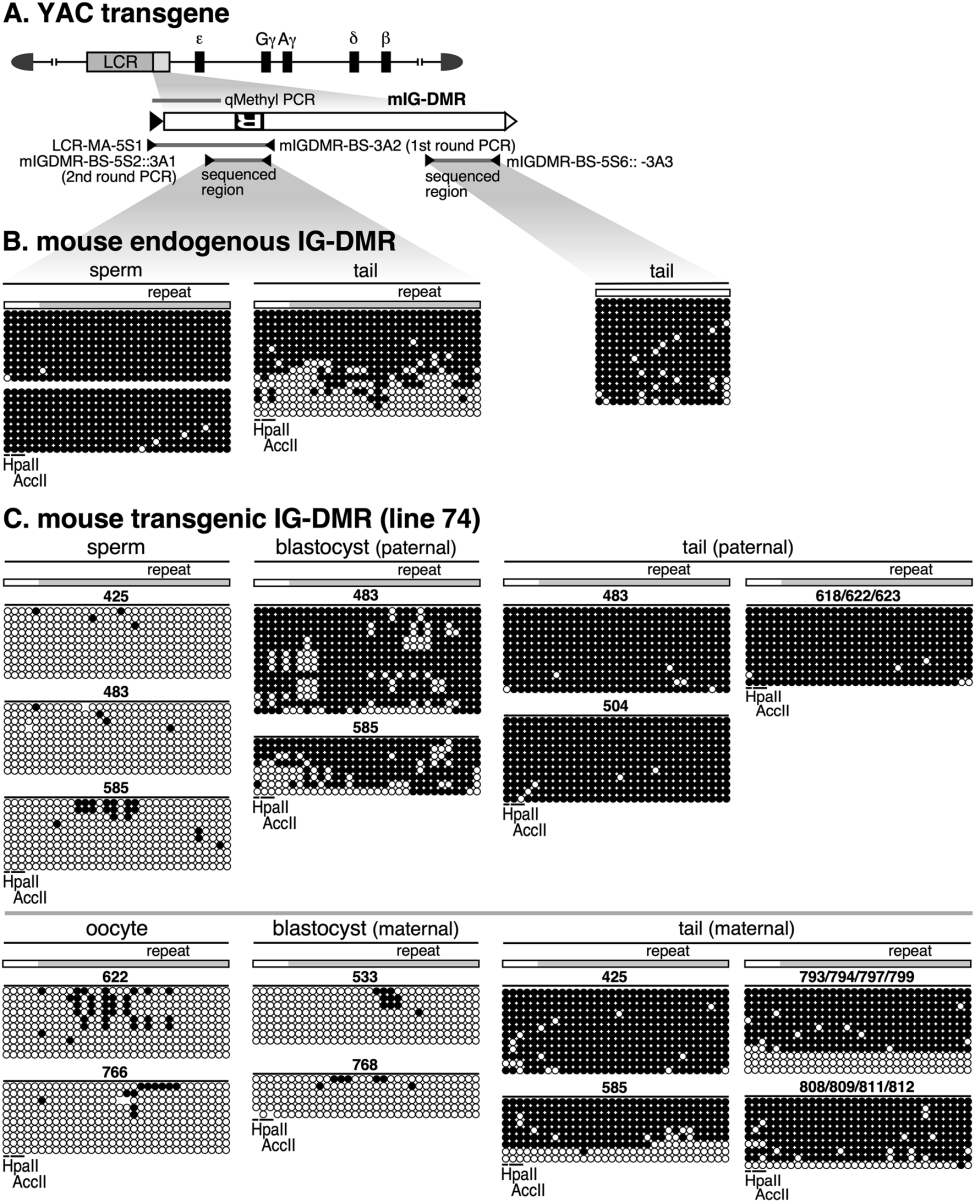

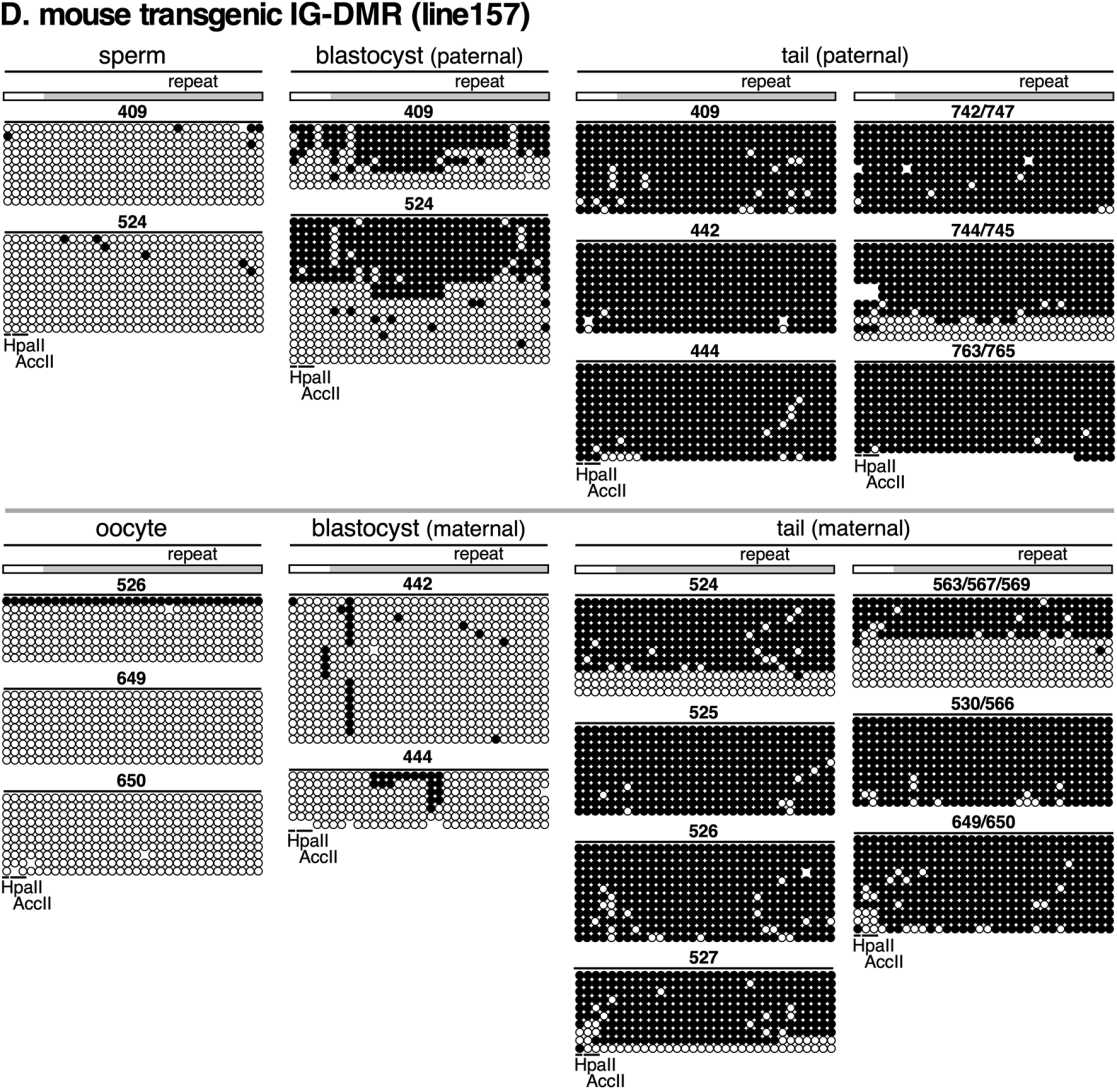
Bisulfite sequencing analysis of mIG-DMR transgene. **A** Enlarged map of the mIG-DMR transgene. The region (gray horizontal bar) analyzed by qMethyl-PCR in Fig. 4 is shown above the enlarged map. The regions amplified by nested PCR for BS sequencing and primer sets used are shown beneath the enlarged map. **B** Genomic DNA from sperm germ cells (two biological replicates) and tail-tip somatic cells of non-Tg mouse was subjected to BS sequencing. Location of repeat sequence is shown by gray rectangle. The CpG motifs in the *Acc*II/*Hpa*II recognition sites are marked by horizontal lines. **C**, **D** Genomic DNAs from germ cells (sperm or oocyte), blastocyst, and tail-tip somatic cells of TgM (lines 74 and 157 in C and D, respectively) inheriting the transgene either paternally (upper panels) or maternally (lower) were subjected to BS sequencing. ID numbers shown above each block correspond to those shown in Fig. 4B, C

Next, we used bisulfite sequencing to analyze the DNA methylation state of the mouse IG-DMR (Fig. 5A). In tail somatic cells (Fig. 5B), while the 5' region of the endogenous locus did not appear to be a DMR, we found a bimodal distribution of hyper- and hypomethylated DNA strands in the region around the repeat sequence, and the same region was methylated in sperm. This observation is consistent with previous reports [35, 36] showing that this region is a paternally methylated DMR.

To distinguish between endogenous and transgenic sequences around the repeat sequence in TgM (Fig. 5A), we performed nested PCR. In both lines 74 (Fig. 5C) and 157 (Fig. 5D), the transgene was not methylated in either sperm or eggs. In blastocyst stage embryos, however, the sequence was preferentially methylated in the paternally inherited transgene, indicating that allele-specific methylation occurred during the post-fertilization period. The lower degree of methylation acquisition in line 157 compared to that in line 74 is likely due to position-effect variegation, although the difference in the methylation magnitude between the two lines was opposite to that observed with the r*H19* ICR transgene. This may be because the DNA methylation sensitivity, which depends on the surrounding chromatin state, is different for each transgene.

After implantation (i.e., in the tail), however, the maternal transgene sequence in both lines became highly methylated, suggesting that the IG-DMR fragment we tested does not protect the maternal IG-DMR from genome-wide de novo DNA methylation after implantation. It is noteworthy that the level of methylation was moderately lower in the maternal sequence (especially outside the repeat sequence) than in the paternal sequence, which enabled us to detect allelic differences in the methylation level by qMethyl-PCR (Fig. 4).

In summary, these results demonstrated that the mouse IG-DMR sequence underwent post-fertilization methylation imprinting, yet the maternal sequence was not protected from post-implantation, genome-wide de novo methylation activity, and eventually, the imprinted DNA methylation state of the sequence became ambiguous. In contrast, the rat *H19* ICR transgene did not undergo imprinted DNA methylation despite the fact that its sequence is highly homologous to that of the mouse *H19* ICR.

## Discussion

We previously reported post-fertilization imprinted methylation phenomena in mouse and human *H19* ICR sequences in TgM [13, 29]. Although these sequences share similar CG-rich sequences, as well as binding motifs for CTCF, Sox/Oct, and Zfp57 transcription factors, their overall structures were not well conserved (Additional file 1: Fig. S1A), which prevented us from identifying a human sequence corresponding to the mouse 118-bp sequence that is essential for post-fertilization imprinted methylation. Therefore, in this study, we used the rat *H19* ICR sequence, which is highly conserved in overall structure (including the relative placement of binding sites for the transcription factors; Additional file 1: Fig. S1A), to generate TgM. Surprisingly, however, no imprinted DNA methylation was observed after fertilization (Figs. 2 and Fig. 3). Since mIG-DMR analyzed at the same genomic location exhibited post-fertilization imprinted methylation, it is unlikely that the results in the rat sequence are related to the insertion site of the transgene. In addition, our previous reconstruction experiments showed differential methylation of the LCb118 fragment, in which the CTCF, Sox/Oct, and Zfp57 motifs were embedded in lambda DNA in the same context as the mouse *H19* ICR and then combined with the mouse 118-bp sequence [28]. Because binding sequences for these factors are highly conserved in number and arrangement between mouse and rat *H19* ICRs (Additional file 1: Fig. S1A, B), it is likely that the rat *H19* ICR (other than the 113-bp sequence) exerts activity comparable to that of the corresponding region of the mouse *H19* ICR. Comparison of the 118-bp mouse sequence and the 113-bp rat sequence revealed that the homology between the two species was not very high (65%). In particular, Zfp57 recognition motif-like (Zfp57-like) sequences are present in the mouse and human *H19* ICR transgenic sequences, but not that in the rat (Additional file 1: Fig. S1A, C). This difference may account for the lack of post-fertilization imprinted methylation activity in the 2.8-kb rat *H19* ICR sequence. If this assumption is correct, unidentified factors that recognize this motif may regulate post-fertilization imprinted methylation, since Zfp57 does not bind to the motif in vitro [27]. Furthermore, if the rat *H19* ICR undergoes post-fertilization imprinted methylation at its native, endogenous locus, then the regulatory sequence may lie outside the 2.8-kb transgene sequence used in this study. It is also possible that interspecies differences in *trans*-factors between mouse and rat may account for the lack of post-fertilization imprinted methylation of the rat *H19* ICR in mice.

Although imprinted methylation was initially established in the mIG-DMR transgene after fertilization, this differential methylation state was lost due to de novo methylation on the maternal allele after implantation. In other words, the 2.7-kb fragment used in this study appears to lack the sequence necessary to protect the maternal sequence from global de novo methylation after implantation. It was recently reported that the IG-DMR (3306 bp) consists of two distinct functional elements: a 5ʹ sequence (at the endogenous locus; IG^CGI^) that is bound by Zfp57 and is important for maintaining the hypermethylation state of the paternal sequence, and a 3ʹ sequence (IG^TRE^) that is important for maintaining the hypomethylation state of the maternal sequence (Additional file 1: Fig. S1D) [37]. The transgenic IG-DMR was de novo methylated after implantation in our experimental setup, possibly because the fragment we used (2,674 bp) does not contain most of the IG^TRE^. It was also reported that IG^TRE^ enhances expression of the *Gtl2* gene (and associated downstream transcripts) in *cis* at the unmethylated maternal allele. At the paternal allele, the gamete-derived, highly methylated state of the IG^CGI^ induces methylation of the neighboring IG^TRE^, resulting in suppression of its enhancer activity and transcriptional repression of the *Gtl2* gene in *cis*. Consequently, Gtl2-DMR, present at the promoter region of this gene, acquires DNA methylation after implantation [26]. According to this model, the master regulator of genomic imprinting in the entire *Dlk1*-*Dio3* domain is paternal IG^CGI^ methylation, which is consistent with the observation by others that DNA methylation at repetitive sequences artificially introduced by epigenome editing spread throughout the IG-DMR in mouse embryonic stem (ES) cells [38]. In the current study, therefore, the activity that introduced de novo DNA methylation into the paternal IG^CGI^ of pre-implantation embryos can be viewed as part of a mechanism that increases the robustness of the imprinting mechanism in the *Dlk1*-*Dio3* domain.

While maintenance of hypomethylation at the maternal *H19* ICR is known to involve CTCF and Sox/Oct factors, the mechanism at the maternal IG-DMR is not fully understood. Analyses of the larger IG-DMR sequences containing the IG^TRE^, as well as truncated IG^CGI^ sequences in our TgM, would allow us to decipher the roles of these sequences in the establishment and maintenance of imprinted methylation of IG-DMR.

Here we showed that the post-fertilization imprinted methylation we previously reported in mouse and human *H19* ICRs also occurred in the mouse IG-DMR. Recently, whole-genome bisulfite sequencing analysis of a mouse pre-implantation embryo revealed that a number of genomic regions were de novo methylated on the paternal allele after fertilization [39]. Thus, post-fertilization DNA methylation that occurs against genome-wide reprogramming activity, may be an essential mechanism ensuring accurate gene expression in the early embryos. On the other hand, a lack of the activity in the rat *H19* ICR, which is highly homologous to the mouse sequence, should help to identify *cis* sequences responsible for the post-fertilization imprinted methylation activity. We anticipate that identification of *trans*-factors that bind to the sequence will advance our understanding of the molecular mechanism of imprinted methylation maintenance, which we hope will lead to a better understanding of the pathogenesis of imprinting diseases [12, 40].

## Conclusions

We showed that the post-fertilization imprinted methylation, which we previously reported in mouse and human *H19* ICRs, also occurred in the mouse IG-DMR. This result suggests that the methylation mechanism is widely shared among species and genes to protect paternal imprinted methylation from reprogramming during the pre-implantation period.

## Methods

### Preparation of the rat H19 ICR fragment

Two DNA fragments (BH and HG) were generated by PCR using the rat genomic DNA (F344/DuCrlCrlj/Charles river) as a template and two sets of primer pairs: Rat-H19ICR-BH-5S, 5ʹ-CAAGggAtcCAGAGCCCTGACTTCTAGTCT-3’ (*Bam*HI site is underlined; artificially introduced nucleotides are shown in lower case letters) and Rat-H19ICR-BH-3A, 5ʹ-GGTGGCAGTACAACCCTACGTATT-3ʹ, or Rat-H19ICR-HG-5S, 5ʹ-GCCTGACCCCTTTGTTGAACCTGG-3ʹ and Rat-H19ICR-HG-3A, 5ʹ-CCTCAGGAgAtCTGAGCTCTTTCTCTACCA-3ʹ (*Bgl*II site underlined). The BH and HG fragments were digested with *Bam*HI/*Hin*dIII and *Hin*dIII/*Bgl*II, respectively, and linked together at their *Hin*dIII ends to generate the rat *H19* ICR fragment (Chr1: 197,734,995–197,737,805; mRatBN7.2; 2811 bp).

### Preparation of the mouse IG-DMR fragment

The mouse IG-DMR fragment (Chr12: 109,526,327–109,529,000; mm10; 2674 bp) was generated by PCR using murine genomic DNA (B6-ES) as a template with the following set of primers: mIG-DMR-5S2, 5ʹ-TGTCAGGAGGACTCTGAGAGATGA-3ʹ and mIG-DMR-3A3, 5ʹ-TTGAagaTcTGAGgGATcCTCAGAAAGGCAGTGGGGGAAG-3ʹ (*Bgl*II and *Bam*HI sites are underlined; artificially mutated nucleotides are shown in lower case letters). The resultant fragment was digested with *Bgl*II.

### Yeast targeting vectors and homologous recombination in yeast

The co-placement target vector, pHS1/loxP-5171-B-2272-5171-G-2272 (pCop5B25G2), carrying a human β-globin HS1 sequence [nucleotides 13,299–14,250 (HUMHBB; GenBank)] in which 5'-loxP5171-*Bam*HI-loxP2272-loxP5171-*Bgl*II-loxP2272-3' sequences are introduced into the *Hin*dIII site [at nucleotide 13,769 in HUMHBB], was reported elsewhere [24].

The mouse IG-DMR fragment was inserted into the *Bam*HI site of pCop5B25G2 to generate pCop-mIGDMR(-). The resultant plasmid was digested with *Bgl*II and ligated with the rat *H19* ICR fragment to generate pCop-mIGDMR(-)/rH19ICR(-). In each cloning step, the correctness of DNA construction was confirmed by DNA sequencing.

The targeting vector was linearized with *Blp*I [at nucleotide 13,455 in HUMHBB] and used to mutagenize the human β-globin YAC (A201F4.3) [41]. Successful homologous recombination in yeast was confirmed by Southern blot analyses with several combinations of restriction enzymes and probes.

### Generation of YAC-TgM

Purified YAC DNA was microinjected into fertilized mouse eggs from C57BL/6J (Charles River) mice. Tail DNA from founder offspring was screened first by PCR, then by Southern blotting. Structural analysis of the YAC transgene was performed as described elsewhere [41, 42]. TgM expressing Cre recombinase in oocytes (Zp3-Cre, Jackson Laboratory, [43]) were mated with parental YAC-TgM lines to derive sublines (i.e., each carrying one of the test fragments). Successful Cre-loxP recombination was confirmed by PCR and Southern blot analyses.

### DNA methylation analysis by qMethyl-PCR

Genomic DNA was prepared from tail-tip cells of 1- to 2-week-old TgM using standard procedures. The DNA methylation level of the transgenic rat *H19* ICR sequence (around the CTCF site 3; Fig. 2A) was determined as follows. Tail somatic DNA was incubated at 60 °C for 3 h in the absence (Reference sample) or presence (Test sample) of *Bst*UI restriction enzyme. The reaction mixtures were diluted and subjected to quantitative PCR using TB Green Premix Ex Taq II (TAKARA, Shiga, Japan) and the following transgene-specific PCR primers: rH19ICR-qMe-5S4, 5ʹ-ACGTCTTACCACCCCTATGAACTG-3ʹ and rH19ICR-qMe-3A3, 5ʹ-ATCAGCCAGTGCGGCTCACTATCA-3ʹ (192-bp amplicon). PCR was performed using the following program: 95 °C for 3 min and [95 °C for 5 s and 58 °C for 1 min] × 45 cycles. The methylation level (%) around the *Bst*UI site of the target sequence was calculated using the following equation: 100 × 2^−∆Ct^, where ∆Ct is determined by subtracting the Ct value of the Reference reaction from that of Test reaction.

The DNA methylation level of the IG-DMR sequence in TgM (Fig. 4A) was quantified using the One-step qMethyl Kit (Zymo Research, Irvine, CA) and the following transgene-specific PCR primers: mIGDMR-qMe-5S1, 5ʹ-TATACGAAGTTATGGATCTGAGGG-3ʹ (part of the loxP sequence is underlined) and mIGDMR-qMe-3A1, 5ʹ-CACAGATTGGGAATGGGATCACGC-3ʹ (447-bp amplicon). The following program was used for restriction enzyme reactions and PCR amplification: 37 °C for 3 h, 95 °C for 30 s, and [95 °C for 30 s, 58 °C for 1 min, and 72 °C for 1 min] × 45 cycles.

### DNA methylation analysis by bisulfite DNA sequencing

Genomic DNA extracted from tail-tip cells or adult sperm was digested with *Xba*I and treated with sodium bisulfite using the EZ DNA Methylation Kit (Zymo Research). Eggs from a super-ovulated female or blastocysts were embedded in agarose beads and treated with sodium bisulfite as described previously [18].

The subregion of each endogenous or transgenic rat *H19* ICR sequence was amplified by PCR using EpiTaq HS (TaKaRa) and the rH19ICR-BS1 primer set: rH19ICR-BS-5S1, 5ʹ-TGTATGTGTTTTGTTTTTTTAGTGAAG-3ʹ and rH19ICR-BS-3A1, 5ʹ-ATCCCAACAAAATCCAATATTCCTA-3ʹ.

The subregion of the mouse IG-DMR transgene was amplified by nested PCR. First-round PCR was conducted using the following transgene-specific mIGDMR-BS-Tg3 primer set: LCR-MA-5S1, 5ʹ-TATAGATGTTTTAGTTTTAATAAG-3ʹ and mIGDMR-BS-3A2, 5ʹ-ACTATAAACCCAAACTACAATTCAC-3ʹ. The following program was used: 94 °C for 5 min and [94 °C for 1 min, 58 °C for 2 min, and 72 °C for 2 min] × 25 cycles. To minimize contamination of the template genomic DNA, the PCR product was diluted 250 fold and used for second-round PCR using the mIGDMR-BS-Com2 primer set common to both endogenous and transgenic sequences: mIGDMR-BS-5S2, 5ʹ-GGGGGGAAATTTTGTAAGTATTAGA-3ʹ and mIGDMR-BS-3A1, 5ʹ-AATACACAAACTAACCATATACAAA-3ʹ. In this experimental condition, the absence of a first-round reaction was associated with almost no PCR amplification in the second-round reaction.

The PCR products were subcloned into the pGEM-T Easy vector (Promega, Madison, WI) for sequencing analyses. Sequencing results were analyzed using the QUantification tool for Methylation Analysis (QUMA, http://quma.cdb.riken.jp).

## Supplementary Information

**Additional file 1: Figure S1.** Schematic representation of the sequence features of rat *H19* ICR and mouse IG-DMR. **A** Comparison of mouse, rat, and human *H19* ICR sequences. The CpG dinucleotides are shown by thin vertical lines with their numbers in parentheses. Positions of CTCF and Sox/Oct binding motifs are shown by solid circle and thick vertical lines, respectively. In the mouse and rat sequences, the118-bp and 113-bp sequences are denoted by gray rectangles. Position of Zfp57 binding consensus and its similar (Zfp57-like) sequences is shown beneath each map. **B** Comparison between mouse and rat consensus CTCF (underlined) and Zfp57 (reversed black and white) binding sequences. Identical nucleotides are denoted by vertical lines. **C** Comparison between mouse 118-bp and rat 113-bp sequences. The Zfp57-like sequences are highlighted and underlined in the mouse sequence. Identical nucleotides are denoted by vertical lines. **D** Characteristic of the mouse *Dlk1-Dio3* genome locus. IG^TRE^ and IG^CGI^ regions are shown [37]. Distribution of the CpG dinucleotides and position of the consensus Zfp57 sequences is shown.

## Data Availability

The datasets used and analyzed during the current study are available from the corresponding author on reasonable request.
