# Supplementary figures and images for: The transgenic IG-DMR sequence of the mouse Dlk1-Dio3 domain acquired imprinted DNA methylation during the post-fertilization period
Source: Epigenetics Chromatin. 2023 Feb 17;16:7. doi: 10.1186/s13072-023-00482-x (PMC9936741; doi:10.1186/s13072-023-00482-x)

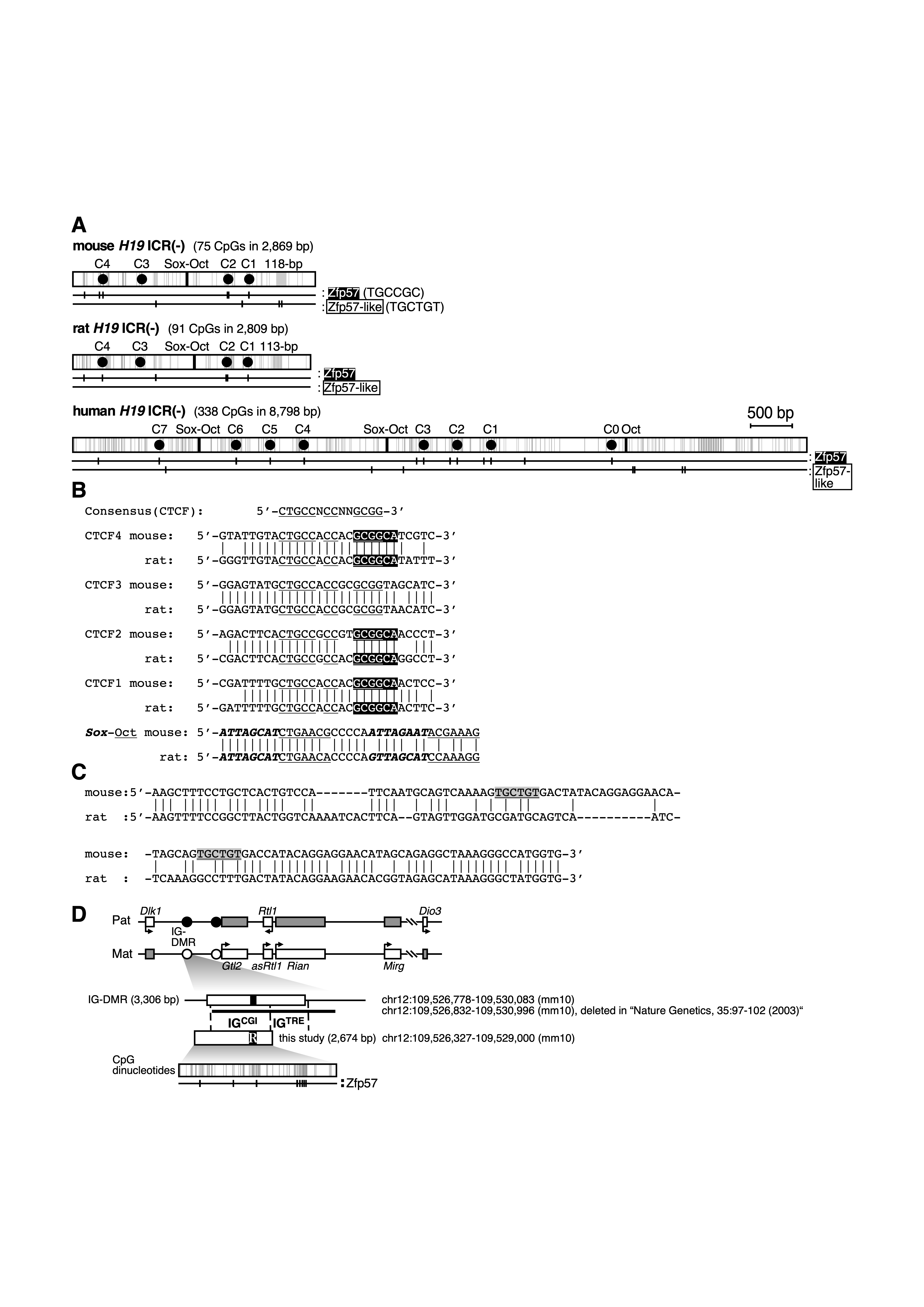

Supplement: Supplementary file 1 — Additional file 1: Figure S1. Schematic representation of the sequence features of rat H19 ICR and mouse IG-DMR. A Comparison of mouse, rat, and human H19 ICR sequences. The CpG dinucleotides are shown by thin vertical lines with their numbers in parentheses. Positions of CTCF and Sox/Oct binding motifs are shown by solid circle and thick vertical lines, respectively. In the mouse and rat sequences, the118-bp and 113-bp sequences are denoted by gray rectangles. Position of Zfp57 binding consensus and its similar (Zfp57-like) sequences is shown beneath each map. B Comparison between mouse and rat consensus CTCF (underlined) and Zfp57 (reversed black and white) binding sequences. Identical nucleotides are denoted by vertical lines. C Comparison between mouse 118-bp and rat 113-bp sequences. The Zfp57-like sequences are highlighted and underlined in the mouse sequence. Identical nucleotides are denoted by vertical lines. D Characteristic of the mouse Dlk1-Dio3 genome locus. IGTRE and IGCGI regions are shown [37]. Distribution of the CpG dinucleotides and position of the consensus Zfp57 sequences is shown. [file 13072_2023_482_MOESM1_ESM.tif]
